# Spatial learning in Japanese eels (*Anguilla japonica*)

**DOI:** 10.1007/s10071-019-01320-y

**Published:** 2019-10-24

**Authors:** Shigeru Watanabe, Kazutaka Shinozuka

**Affiliations:** 1 Department of Psychology, Keio University, Mit 2-15-45, Minato-Ku, Tokyo, Japan; 2 RIKEN Center for Brain Science, 2-1 Hirosawa, Wako, Saitama Japan

**Keywords:** eel, Spatial cognition, Discrimination, Global cue

## Abstract

Japanese eels (*Anguilla japonica*) were trained on a Morris-type spatial learning task. There were four tubes in a pool, but the eels could hide in only one of these. The eels learned the position of the open tube, and maintained their performance when the pool was rotated to remove possible intra-maze cues. The eels could not maintain their performance in a dark room, suggesting that spatial learning involved extra-maze visual cues. When the position of the open tube was randomly changed every day, the performance of the eels in finding the open tube did not improve.

## Introduction

Fish have remarkable orientation and navigating abilities during migration (Dodson 1988). Fish living in complex environments may need spatial memory capabilities. Aaronson (1971) constructed an artificial tide pool, and confirmed that jumping gobies (*Bathygobius soporator*) learned the configuration of the environment during high tide, and used this map to jump to the safe tide pools during the artificial low tide.

The spatial memory of fish has also been experimentally studied in the laboratory. A variety of apparatus developed for rodents has been applied to fish. One classic apparatus is the T or Y maze (Zerblio and Wickstra 1980; White and Brown 2015). A plus maze is also used (McAroe et al. 2016). The radial arm maze has been widely used in experiments with rodents, but radial maze experiments using fish are rare (Roitblat et al. 1982). Another standard apparatus designed for rodents is the Morris water maze. We developed a new spatial learning task for goldfish (*Carassius auratus*) that was comparable to a dry version of the Morris maze (Saito and Watanabe 2005).

Among fish, eels have an outstanding migratory ability. Tsukamoto et al. (2011) found hatched eggs and larvae of Japanese eels in the western Mariana region. Thus, hatched larvae migrate to Japan, and adult eels swim thousands of kilometers back to the western Mariana region. Eels should have a high spatial cognition ability in nature, but their spatial cognition has never been examined in the laboratory. Here, we created a Morris maze-like apparatus for eels. The eels preferred to hide in a long small hole, such as a tube, and we used this behavior to train the eels on spatial memory tasks. Four tubes were arranged in a circular water tank, and one of these was open so that the eels could enter it. Thus, the eels could learn the task as escape learning, similar to the original Morris water maze. After the eels completed the tasks, the cues used by the eels for learning were examined.

## Methods

### Study subjects

Seventeen Japanese eels (*Anguilla japonica*) obtained from Omori-Tansui, Miyazaki Japan, were used. The total length of the eels was 22–35 cm. The eels were housed individually in aquaria (39.8 × 25.4 × 28 cm), and each aquarium had an air pump. Sand was placed on the floor of each aquarium, and a gray vinyl chloride tube (inner diameter: 1.5 cm; length: 24 cm) was added to each aquarium. The experiments started 2 weeks after the eels arrived at the laboratory. A 13L:11D artificial illumination cycle was used, but the racks for the aquariums were covered by a gray vinyl curtain.

### Apparatus

**Fig. 1:**
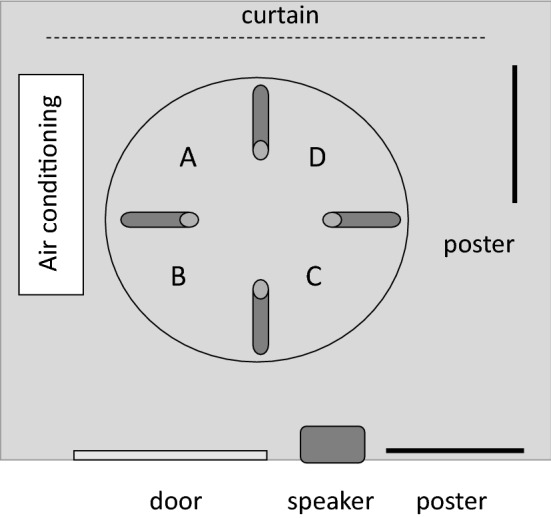
Experimental pool. The eels were randomly released from four different positions (*A*–*D*)

The experimental maze consisted of a white polypropylene circular pool with a diameter of 100 cm and a depth of 38 cm (see Fig. 1). The water level was 5 cm from the bottom of the pool. The water temperature was kept at 25 °C, and the water was changed every fifth day. The experimental room was illuminated with fluorescent lamps, and there were several extra-maze cues in the room (see Fig. 1a). The pool contained four gray vinyl chloride tubes (inner diameter: 1.5 cm; length: 24 cm), and each tube had a lead weight attached to it to fix it on the floor. A transparent acyl cylinder was inserted into three of the four tubes, so that eels could not enter the tubes. The other tube was left open so that the eels could enter it. The behavior of the eels was monitored using a CCD camera (G100, NEC Avio) connected to a computer. A night scope (Super Night Compact 1000 NDX; Kenko Tokina Co. Ltd., Tokyo, Japan) was used to observe the eels in the dark room.

### Experimental procedure

#### Habituation to the apparatus

Each eel was individually transferred from its aquarium to a carrying bucket with a nylon net. Each eel was gently released from the bucket into the pool. During the habituation phase, all the tubes were open, and the eels were allowed to move around the pool for 10 min. Usually, the eels selected one tube and stayed inside it. After 10 min, the tube was picked up and the eel was slid back down into the net, and returned to the aquarium. All tubes were cleaned with a brush. Then, the next eel received the habituation training. This procedure was repeated for 2 days. Another group of eels (control group) received similar habituation.

#### Experimental group

Twelve eels were used for spatial discrimination. During the spatial discrimination phase, the treatment of the eels was identical to that during the habituation phase, except only one tube at a fixed position was open. If an eel entered the open tube, it could stay there for 10 min. If an eel did not enter the tube, it was retrieved after 10 min. The eels underwent just one training trial per day, and the position of the release was randomly changed (see Fig. 1). The criterion of discrimination was three correct responses within four successive trials. However, the eels underwent at least ten training trials. After the eels met the criterion, they underwent the following test.

#### Rotation test

The eels might use unknown visual cues inside the pool to learn which is the correct tube. The pool was rotated 90°, 180°, or 270° to eliminate such intra-maze cues. That is, each eel underwent one trial each at 90°, 180°, and 270°. The tube at the original position, regardless of rotation, was the open tube. Again, each eel underwent one trial test in 1 day.

#### Dark room test

To eliminate extra-maze visual cues, the test was carried out in a dark room. The test procedure was the same as that for the discrimination training, except for the darkness of the room. There was slight leak of illumination, and we measured illumination at nine positions inside the tank. The mean illuminance was 0.10 Lx in the dark room, while it was 368 Lx in the light room. The test was repeated four times, with one trial per day.

#### Control group

Five eels were used as the controls. The training procedure was similar to that for the spatial discrimination group, except that the position of the open tube was randomly changed every day. The training continued for 24 trials. After the training, the eels underwent the dark room test.

#### Statistics

We conducted a sample *t* test to evaluate the performance of the eels. Chance level to enter the correct tube was 0.25 per trial. Thus, chance level of cumulative number of correct trials for the rotation test (three trials) was 0.75, and that for the dark room (four trials) was 1.0. We compared latencies in the rotation and dark room tests with the two-tailed paired *t* test.

## Results

**Fig. 2:**
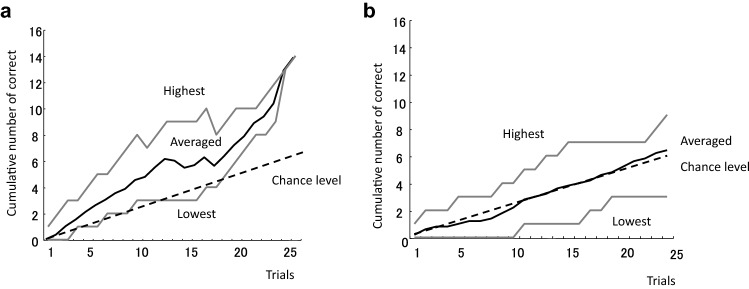
Forward learning curves. The vertical axis indicates the cumulative number of correct trials. ‘Highest’ and ‘Lowest’ indicate the highest and lowest score at each trial. Eels that met the criterion (three correct responses in four consecutive trials) were not considered while averaging the calculations of further cumulative correct trials

Figure 2a shows the averaged forward learning curve of the experimental group expressed by cumulative number of correct trials. Because eels that showed discrimination did not receive further discriminative training, the number of eels decreased in the later trials. The fastest eel met the criterion by ten trials, and the slowest eel met the criterion by 26 trials (average: 16.4 trials). The black line indicates the average, the broken line indicates the expected cumulative number, and gray lines indicate the highest and lowest number at each trials. Figure 2b shows similar average cumulative number of correct trials for 24 trials in the control group. The average cumulative number of correct trials was almost the same as that of the chance level. The mean number of correct trials in the first half and last half was 0.25 and 0.27, respectively. The paired two-tailed *t* test revealed no differences between the first and last halves with respect to the correct response (*t*(4) = 0.23, *P* = 0.83).

**Fig. 3:**
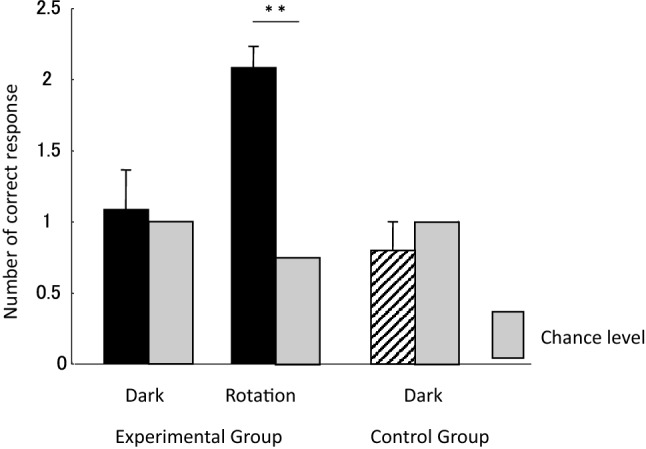
Results of the tests. Gray bars indicate the chance level in each test. **P < 0.001

Figure 3 presents results of the tests. In the experimental group, there was a significant difference from the chance level (0.75) in the rotation test (two-tailed one sample *t* test, *t*(22) = 8.97, *P* < 0.001), but no significant difference from the chance level (1.0) (*t*(22) = 0.29, *P* = 0.77) in the dark room test. These results indicate that spatial learning was based on visual extra-maze cues in the eels. The control group showed chance level performance in the dark room test. There was no significant difference from the chance level (two-tailed one sample *t* test, *t*(5) = 1.0, *P* = 0.37).

## Discussion

The present results demonstrate (1) the eels’ ability to learn the spatial memory task, (2) the usefulness of a tube shelter as a behavioral reinforcer, and (3) spatial learning in eels is based on visual extra-maze cues. The results of the control group demonstrated that the eels could not discriminate the open tube on the basis of non-spatial cues, such as the presence of the blocking cylinder.

Due to procedural differences, precise comparison between our results in eels and those found in studies using other fish is rather difficult, but goldfish learned the Morris-type maze in approximately 12–16 trials (four trials per session) (Saito and Watanabe 2005), whereas the eels learned the task by on average 16.4 trials. Thus, the spatial learning ability of the eels is approximately similar to that of the goldfish. The effectiveness of behavioral reinforcement by hiding into a shelter tube means that this training method can be applied in a variety of studies, because eels can survive for 1 year without food.

Omura et al. (1997) observed cones in the retina of larvae of Japanese eels, and Byzov et al. (1998) reported yellow-sensitive and green-sensitive cones in European eels (*A. anguilla*). Interestingly, eels show changes in morphology at the time of downstream migration. Changes in skin color, degeneration of the gut, and changes in fat and musculature have been reported. Increases in eye size have also been reported. Hagiara et al. (2012) reported enlargement of the eyes in migrating tropical eels in comparison to non-migrating ones. Artificial maturation by hormone injection in female eels caused enlargement of the eyes, an increase in the number of rods, and a decrease in the number of cones (Pankhurst 1982). However, electroretinographs (ERG) showed no change in scotopic sensitivity by artificial maturation (Pankhurst and lythgoe 1983). The significance of the enlargement of the eye is not yet known, but one possibility is that it plays a role in setting up the visual system for migration. Animals use different sensory modalities for navigation, and visual cues provide them with a lot of spatial information. The present study found that the visual system plays a role in spatial cognition in a small space, suggesting that it is possible that visual cognition also plays a role in visual cognition in larger spaces.

Eels are easy to keep in a laboratory, and may be used as a possible experimental animal for studies on animal spatial cognition.
